# Successful endoscopic surgical treatment of pleuroperitoneal communication in two infant cases

**DOI:** 10.1186/s40792-021-01266-9

**Published:** 2021-08-12

**Authors:** Teizaburo Mori, Akihiro Fujino, Masataka Takahashi, Ryoya Furugane, Tamotsu Kobayashi, Motohiro Kano, Akihiro Yoneda, Yutaka Kanamori, Ryutaro Suzuki, Kentaro Nishi, Koichi Kamei, Masayuki Kitamura

**Affiliations:** 1 Division of Pediatric Surgery, National Center for Child Health and Development, 2-10-1 Okura Setagaya-ku, Tokyo, 157-8535 Japan; 2 Division of Nephrology and Rheumatology, National Center for Child Health and Development, Tokyo, Japan; 3 Division of Radiology, National Center for Child Health and Development, Tokyo, Japan

**Keywords:** Pleuroperitoneal communication, Peritoneal dialysis, Pleural effusion, Video-assisted thoracoscopic surgery, Infant, Indocyanine green

## Abstract

**Background:**

Pleuroperitoneal communication (PPC) is an uncommon, but potentially life-threatening complication of peritoneal dialysis (PD). If a fistula does not close with conservative treatment, surgical repair is required. However, approximately half of these patients are forced to shift from PD to hemodialysis. Although it is important to confirm the site of the fistula to achieve a successful surgical treatment, this identification is more difficult in pediatric patients than in adults.

**Case presentation:**

We report two infantile cases of severe PPC associated with PD. In both cases, the age at onset was less than 2 years, and right-sided pleural effusion with dyspnea was observed. PPC was diagnosed by the change in color of the pleural fluid after the injection of a dye into the peritoneal cavity. Peritoneal scintigraphy and single-photon emission computed tomography and computed tomography (SPECT/CT) were performed, and these were effective in locating the fistula site. Endoscopic surgery (video-assisted thoracic surgery (VATS) and laparoscopic surgery) was performed. Indocyanine green (ICG), which was injected into the abdominal cavity, showed the exact site of the fistula. The fistula was successfully closed by attaching an absorbable sheet to it from the thoracic side and an autograft (the falciform ligament) to it from the abdominal side in one patient. In the other patient, the fistula site was resected and sutured, and reinforced with an absorbable sheet. In both cases, PD was resumed without any complication.

**Conclusion:**

We successfully treated two infants of PPC by endoscopic surgery. To identify the fistula site, the ICG navigation method was useful. Even in small infants, PPC can be treated successfully by endoscopic surgical repair if the site of the fistula is identified.

## Background

Pleuroperitoneal communication (PPC) causing massive pleural effusion is an uncommon, but well-known complication among the nephrologists who treat renal insufficiency, and it is potentially life-threatening complication of peritoneal dialysis (PD) [1–3]. Although pleural effusion occurs shortly after PD induction in most cases, it can develop at any time during PD treatment [4]. Pleural effusion in PPC is seen mostly in the right-sided thoracic space [1] and caused by the leakage of peritoneal fluid into the pleural cavity via pleuroperitoneal fistulas [3]. Currently there is no definitive way to accurately identify the fistula [5]. In the past reports, approximately half of these patients are forced to shift to hemodialysis [1, 6] due to the failure to close the fistula.

In pediatric cases with end-stage renal disease, PD is a well-established, preferred and safety method to maintain dialysis therapy [1, 7]. Therefore, it is very important to obtain early diagnosis and grapple with proper treatment for PPC. If the fistula does not close with temporary cessation of PD, a surgical approach is required. However, surgical repair often fails because the site of the fistula is usually very difficult to identify in pediatric cases.

In this report, we present two infants with PPC, for whom surgical treatment was successfully performed by identifying the fistula site using currently available methods. To the best of our knowledge, Case 1 is the first reported case where the fistula site was identified from the abdominal side, and Case 2 is the youngest and the smallest patient with PPC in literature.

## Case presentation

### Case 1

**Fig. 1:**
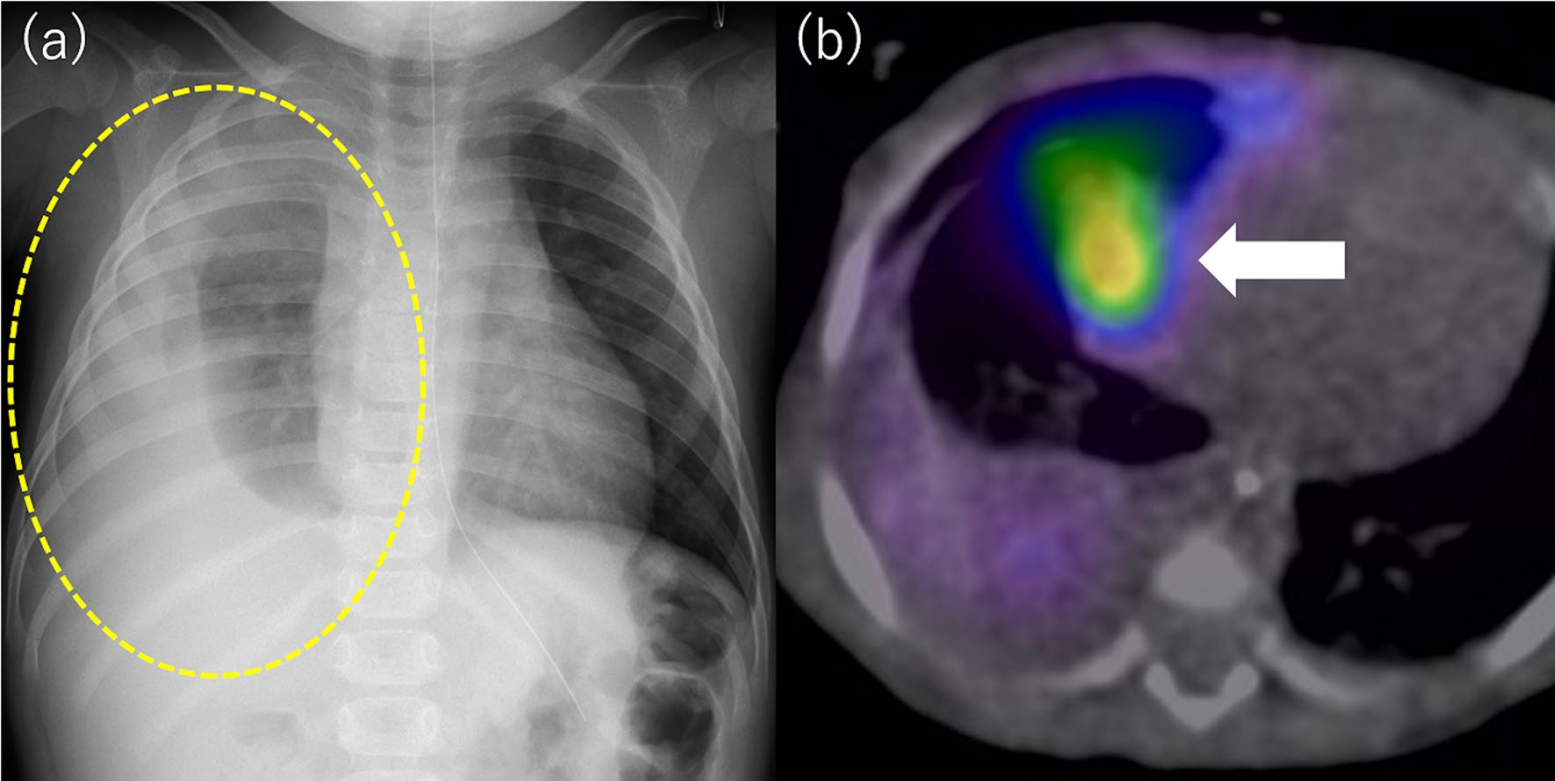
**a** A chest radiograph showed a right-sided hydrothorax. **b** Preoperative scintigraphy demonstrating the radioactive dialysate in the thoracic cavity confirming a PPC. The arrow indicates hot spot which remained for a next day

A 1 year and 10-month-old girl with Denys–Drash syndrome, weighing 10 kg, was referred to our institute with subacute onset of respiratory distress due to the right-sided pleural effusion (Fig. 1a). She was on PD from 5 months of age. She presented with complaints of dyspnea, following decrease in PD fluid drainage for a month. Artificial ventilation support was started due to severe respiratory failure and a chest tube was placed to drainage the right-sided pleural effusion. When indigo carmine dye (20 mg/L) was infused into the abdominal cavity through the PD catheter, the dye color was not detected in the drained pleural fluid under the mechanical ventilation. However, when a re-trial was performed under spontaneous respiration after extubation, blue-colored discharge was found in the chest tube, confirming the diagnosis of PPC. PD was discontinued, and hemodialysis was initiated as a conservative treatment for PPC. Since PPC did not resolve after cessation of PD for 3 months, surgical treatment was performed.

Before the surgery, scintigraphy and single-photon emission computed tomography and computed tomography (SPECT/CT) were performed. Ten minutes after 99 m-Tc-Sn-colloid injection into the peritoneal cavity with dialysate, radioactivity was detected in the right pleural cavity. A hot spot remained in the medial area of the central tendon of the right diaphragm and mediastinal angle until the next day (Fig. 1b).

**Fig. 2:**
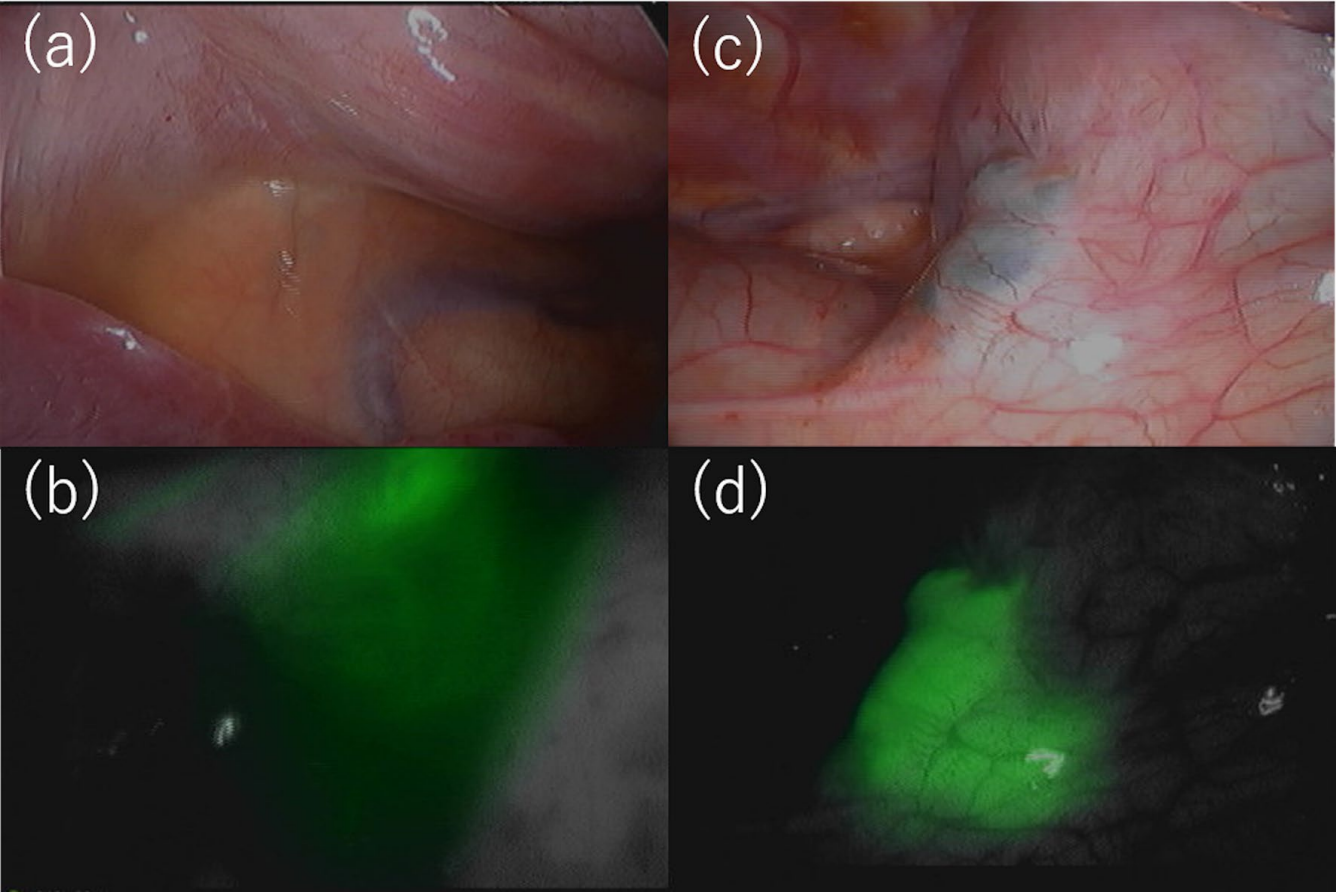
Thoracoscopic findings. **a** The pleural fluid was not stained, but an infrared camera detected fluorescence in the pleural effusion (**b**). A cystic lesion was observed in which the pleura was thinned and dialysate was accumulated under the pleura at the central tendon of the diaphragm of the mediastinal angle (**c**, **d**)

**Fig. 3:**
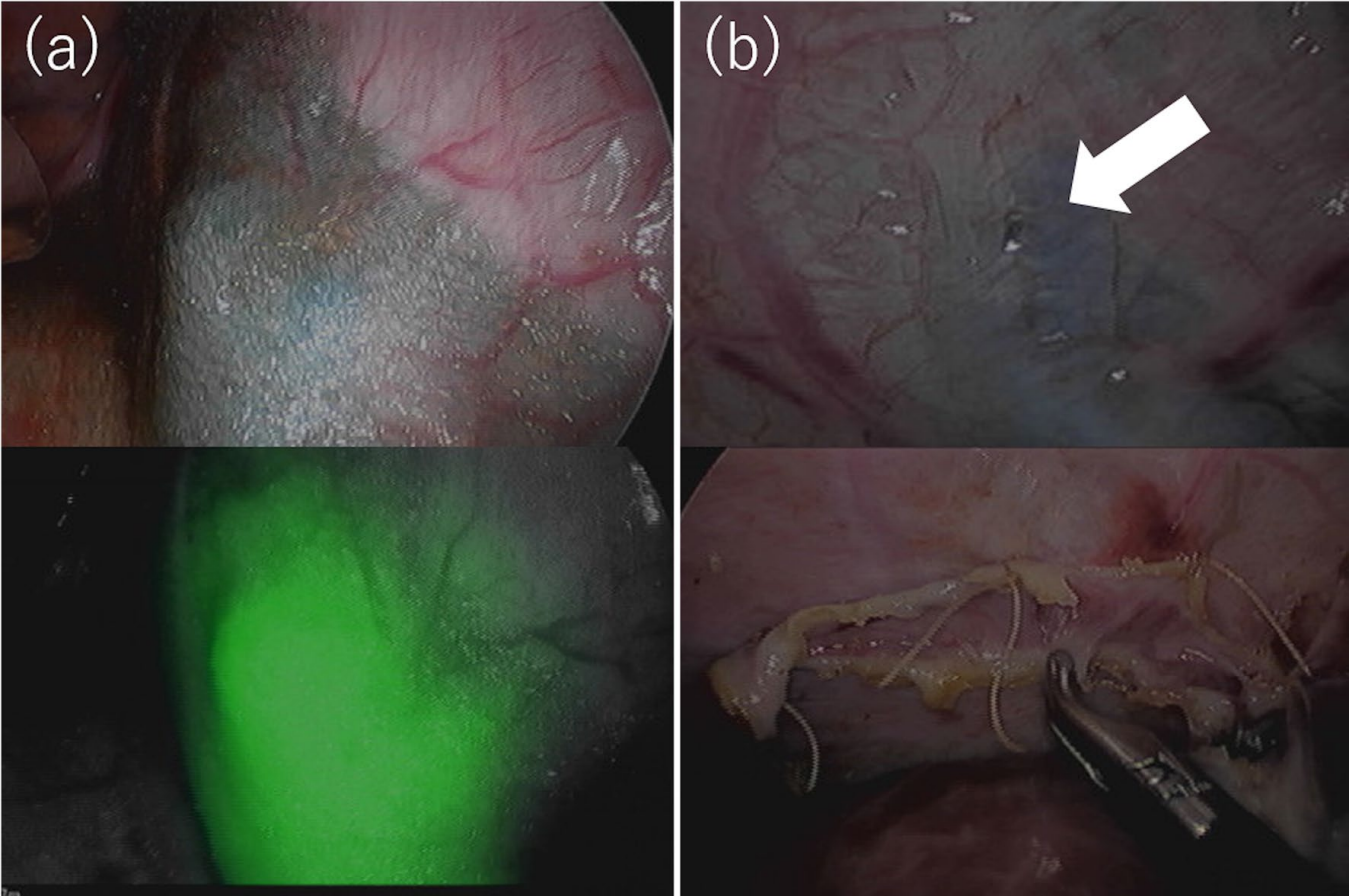
After treatment findings. **a** After scratching the pleural surface, a sheet of absorbable polyglycolic acid felt was attached onto the cystic lesion. **b** The hole (arrow) was covered by suturing a falciform ligament flap patch

For definitive treatment, thoracoscopic surgery under separate lung ventilation was performed, with the patient placed in a left-sided semi-lateral decubitus position. A dialysate containing indigo carmine (20 mg/L) and indocyanine green (ICG) (0.5 mg/kg/L) was injected into the abdominal cavity to identify the leakage site. Though the pleural fluid was not stained blue, an infrared camera detected fluorescence signal in the pleural effusion (Fig. 2a, b). A cystic lesion with thinned out pleura was noted at the site where the hot spot was observed by scintigraphy, and dialysate accumulated under that site (Fig. 2b, c). After scratching the pleural surface, a sheet of absorbable polyglycolic acid felt (Neoveil; Gunze Medical; Tokyo, Japan) was attached to the lesion (Fig. 3a). Following thoracoscopic surgery, laparoscopic PD catheter replacement was performed, and a small hole was found on the peritoneal side of the hot spot. It was thought that the dialysate had likely entered the diaphragm through this hole, after scratching the peritoneal surface, a falciform ligament flap patch was sutured on the hole (Fig. 3b). The patient’s postoperative course was uneventful. The patient resumed PD 2 weeks after surgery, and no recurrence of PPC was observed for 10 months.

### Case 2

**Fig. 4:**
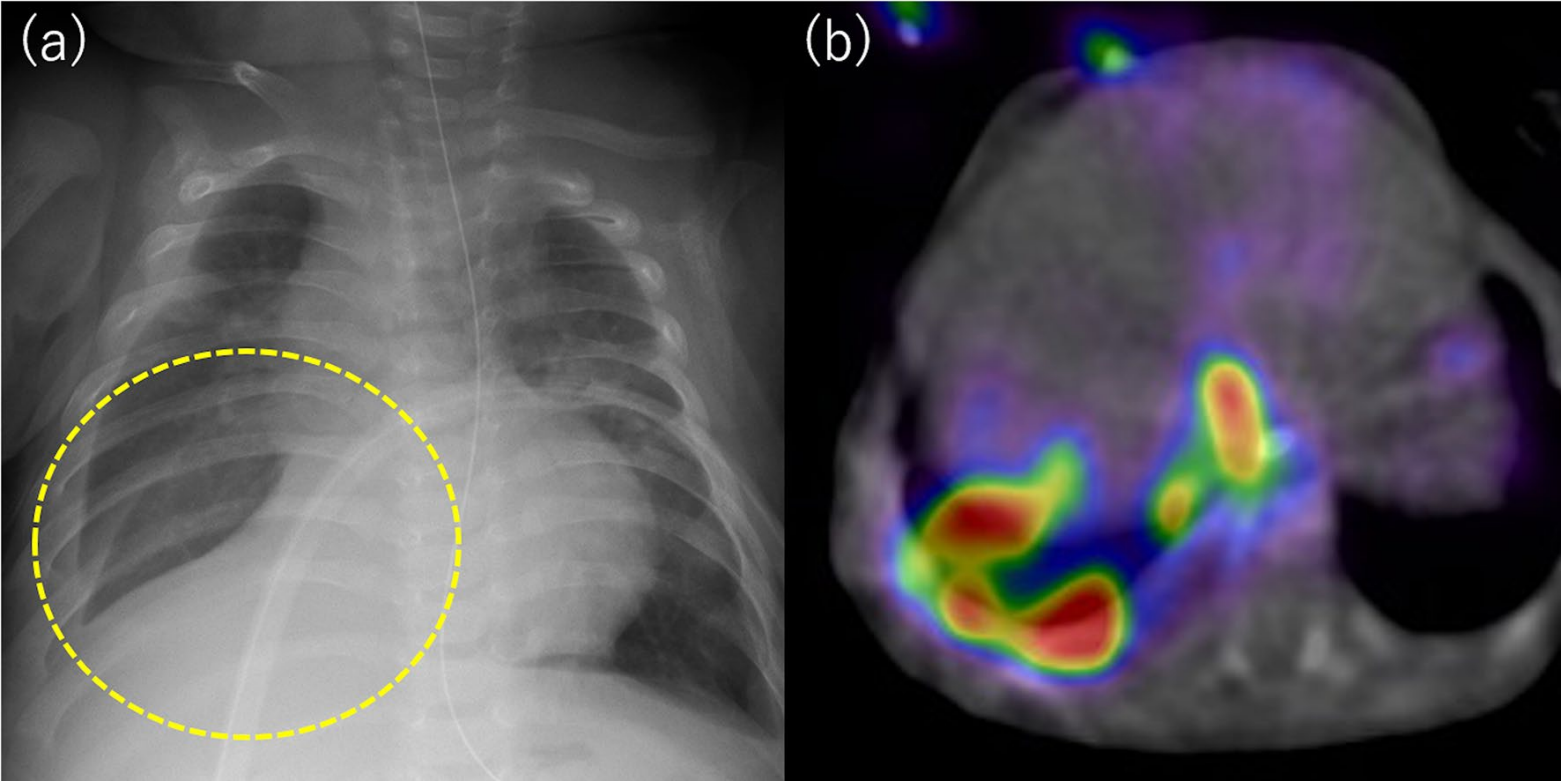
**a** A chest radiograph showed a right-sided hydrothorax. **b** Preoperative scintigraphy demonstrating the radioactive dialysate in the thoracic cavity confirming a PPC

A 5-month-old boy with Potter syndrome, weighing 6 kg, was admitted to our institute with subacute respiratory distress due to right-sided pleural effusion (Fig. 4a). He was on PD from 2 weeks of age. Artificial ventilation support was started, as the pleural effusion worsened after admission, and a chest tube was placed. Indigo carmine dye (20 mg/L) was infused through the PD catheter after positive pressure ventilation support was completed, and blue-colored discharge was found in the chest tube after 5 min, confirming the diagnosis of PPC. PD was discontinued, and hemodialysis was initiated as a conservative therapy. Scintigraphy and SPECT/CT detected leakage of the dialysate into the right pleural cavity, but no remarkable hot spots were observed (Fig. 4b). Since PPC did not resolve spontaneously after 3 months of withholding PD, surgery was attempted.

**Fig. 5:**
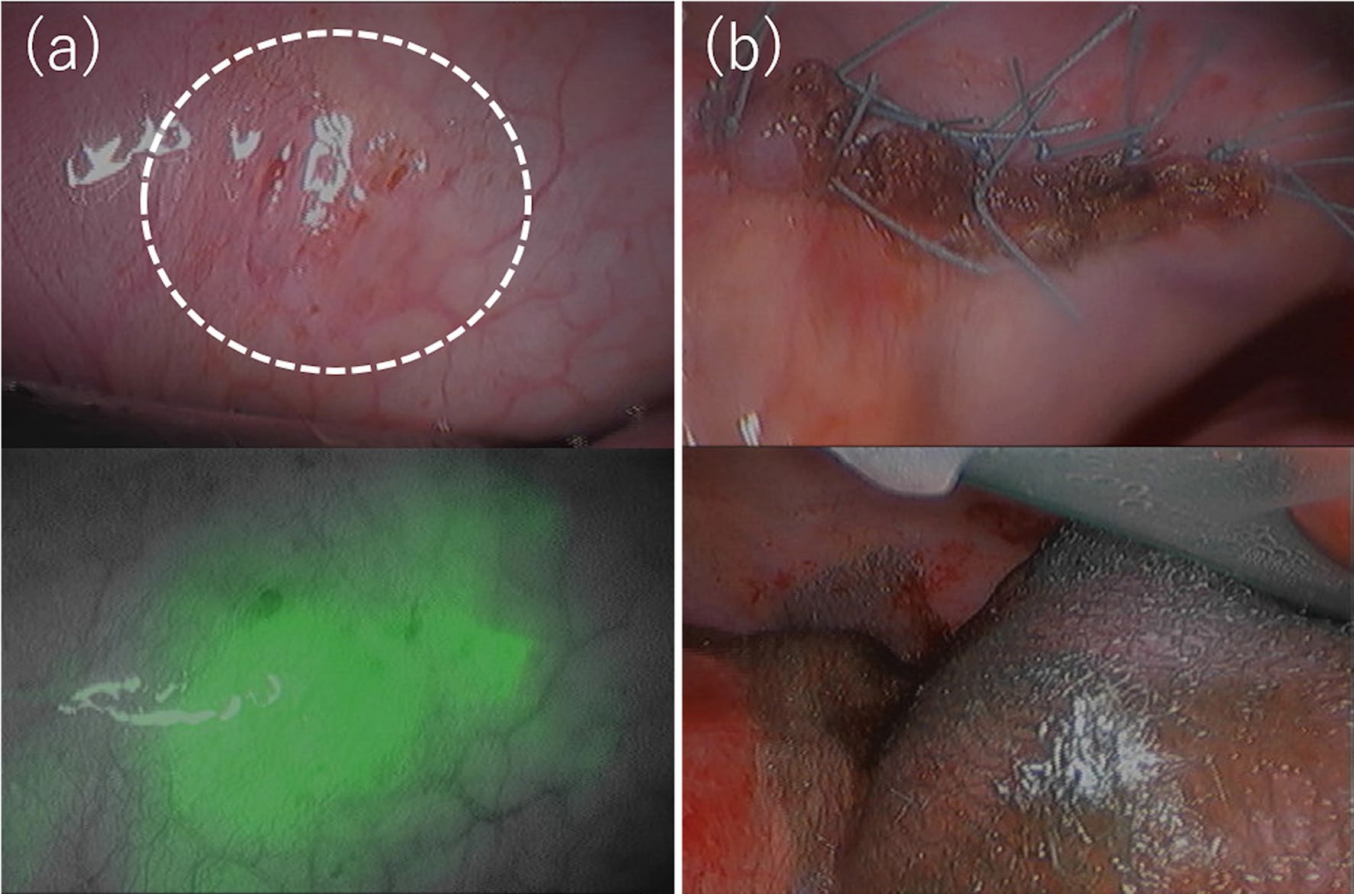
Thoracoscopic findings. The pleural fluid was not stained, but an infrared camera detected fluorescence in the pleural effusion. **a** Small pores were detected. The fluorescence pleural effusion leaked from this site. **b** The lesion was excised and sutured directly, and a sheet of absorbable polyglycolic acid felt was fixed with fibrin glue onto the sutured lesion

Dialysate containing indigo carmine (20 mg/L) and ICG (0.5 mg/kg/L) was injected into the abdominal cavity, and the entire thoracic cavity was inspected carefully by thoracoscopy under separate lung ventilation. Although the pleural fluid was not stained, an infrared camera detected leakage of fluorescent fluid in the lateral edge of the central tendon of the right diaphragm, where small holes were present (Fig. 5a). The diaphragm lesion including the small holes was excised and sutured directly via a small thoracotomy. A sheet of absorbable polyglycolic acid felt (Neoveil; Gunze Medical; Tokyo, Japan) was fixed with fibrin glue onto the sutured lesion (Fig. 5b). The patient’s postoperative course was uneventful. The patient resumed PD 3 weeks later, and no recurrence of PPC was observed during follow-up for 6 months.

## Discussion

Pleural effusion due to PPC in patients undergoing PD was first described in 1967 by Edward and Ungar [8], and its incidence rate was reported 0.64 to 5.1% in adults [1, 6] and 0.9 to 3% in children [1, 9, 10]. At our institute, we experienced two cases of PPC among 101 PD patients in the last 17 years (approximately 2%), which incidence rate was compatible to previous reports.

The exact mechanisms of developing PPC during PD are not well understood, but it is believed that abnormality of diaphragm and/or breakdown of lymphatic network lead to the pleural effusion [11–13]. Anatomical defects are more common in the right diaphragm, and there is a more abundant lymphatic network on the right side than on the left [1]. The lateral edge of the central tendon has abundant lymphatic stomata [14]. These factors may explain the preponderance of right-sided diaphragmatic fistula.

The definitive diagnosis of PPC can be made by the color change of pleural fluid after the injection of dialysate containing a dye such as indigo carmine or ICG into the abdominal cavity through the PD catheter, and/or the scintigraphy [1, 4]. If the patient is under artificial ventilation, we must be careful not to misdiagnose the existence of PPC because the resultant non-physiological pressure imbalance can cause the false-negative result for the diagnosis, as in our cases.

Treatment options include conservative management and surgical repair. Only 58% of the patients achieve long-term PD therapy without recurrence of pleural effusion [6].

Conservative treatments include spontaneous closure of the fistula by withholding PD [1, 4] and chemical pleurodesis [2]. In our cases, we withheld PD for 3 months, as suggested by previous reports [15, 16]; however, this strategy was ineffective. Chemical pleurodesis therapy was not selected because of its low success rate and the possibility of inducing adhesions, which results in difficulty with the surgical approach.

Surgical repair is done by the either technique, thoracotomy or video-assisted thoracoscopic surgery. In 1984, Pattison et al. [17] reported the first case of surgical treatment using a Teflon patch for fistula closure and reported that if the fistula site could not be clearly identified, the success rate dropped to 38% and there was relatively high recurrence rate. In 1996, Di Bisceglie et al. [18] reported on the usefulness of VATS. Since then, VATS has become the standard procedure for the disease in adult patients, with a success rate of approximately 90%, and approaching 100% when specific lesions such as cysts or flaws have been confirmed [19, 20].

We selected VATS technique for our patients although they were under 2-year-old infants because it allows for a thorough observation of the lesions during surgery. To identify the fistula site, a dialysate containing indigo carmine and ICG was injected into the abdominal cavity through a PD catheter. By using these assistive procedures, the lesion could be identified and treated successfully. Among the dyes, only ICG was detected in the operative field. The sensitivity of the infrared camera for fluorescence might be much higher than that of the endoscopic visible light. Thus, we recommend that ICG would be preferred as an aid during surgery.

In case 1, a laparoscope was inserted for PD catheter replacement, and a small hole was identified on the peritoneal surface of the diaphragm. Since direct suturing could tear fragile tissue and an absorbable polyglycolic acid felt could cause undesirable adhesions and reduce dialysis efficiency, autologous tissue (the falciform ligament) was used for patch closure. It may be useful to observe the abdominal cavity when the communication site cannot be identified by thoracoscopy.

## Conclusion

We report two infantile cases of severe PPC associated with PD. Using endoscopic surgery and intraoperative visualization techniques (ICG navigation), we confirmed the critical communicating site and successfully closed the fistula. Even in very small infants, surgical treatment to PPC is effective, by identifying the fistula site using currently available methods.

## Data Availability

All related data are included with the article.

## Abbreviations

ICG: Indocyanine green
PD: Peritoneal dialysis
PPC: Pleuroperitoneal communication
SPECT/CT: Single-photon emission computed tomography and computed tomography
VATS: Video-assisted thoracic surgery
